# Letter to the editor regarding “Effectiveness of injected versus topically applied platelet-rich plasma in diabetic foot ulcers: A randomized controlled trial with clinical and histopathological assessment”

**DOI:** 10.1016/j.jpra.2025.10.032

**Published:** 2025-10-30

**Authors:** Xiuli Li, Yanwei Sun

**Affiliations:** Department of burns & plastic surgery, Zibo Central Hospital, 54# Gong Qing Tuan Xi Road, Zibo, 255036 Shandong Province, PR China

*Dear Sir,*

We read with great interest the article entitled “Effectiveness of injected versus topically applied platelet-rich plasma in diabetic foot ulcers: A randomized controlled trial with clinical and histopathological assessment” by Nasr M, et al.^1^ This study compared the clinical and histological efficacy of injected platelet-rich plasma (PRP) versus topically applied PRP and traditional wound care in the management of diabetic foot ulcers (DFUs). The findings demonstrated that PRP treatment, whether injected or topically applied, significantly enhanced the healing of DFUs compared to traditional wound care. However, several questions remain to be addressed.

Firstly, cutaneous hypoxia and ischemia are the common physiological endpoints of DFUs, often resulting from peripheral vascular stenosis or occlusion.^2^ Ischemia is widely considered the most critical factor impeding DFUs healing. Assessing the anatomical location, morphology, and extent of vascular lesions is essential for guiding treatment decisions. Studies have shown that the healing rate of foot ulcers has significantly improved following direct or indirect vascular reconstruction surgery. Since varying degrees of peripheral vascular lesions can affect DFUs healing, Color Doppler ultrasound is commonly used to measure the diameter of dorsal foot vessels, thereby assessing lower limb vascular lesions. Additionally, parameters such as the ankle-brachial index (ABI), toe-brachial index (TBI), and transcutaneous oxygen pressure (TcPO₂) are recorded to further evaluate vascular status. Therefore, if the authors had measured, recorded, and compared these parameters across the study groups, their conclusions would have been more compelling.

Secondly, the DFUs represents one of the most severe complications of diabetes mellitus, with approximately one-third of diabetic patients at risk of developing DFUs-related infections. The onset of DFUs is closely associated with factors such as peripheral vascular disease and infections. Once ulcers become infected, the affected area and depth tend to expand, and the infection may even spread to the bone. This progression is frequently accompanied by polymicrobial infections, posing significant challenges to clinical management. Although PRP offers advantages in mitigating the inflammatory response when treating DFUs, the efficacy of antimicrobial agents remains critical to the success of diabetic foot therapy. However, for patients with DFUs classified as Wagner grade 2 or higher, antibiotics alone are insufficient to halt disease progression. In current DFUs management, most clinicians prioritize surgical resection of infected tissue or bone, supplemented by systemic antibiotic therapy during the perioperative period. Notably, effective control of wound infection can significantly promote DFUs healing. To robustly evaluate treatment outcomes, blood inflammatory markers (such as white blood cells (WBC), C-reactive protein (CRP) levels), bacterial culture results, and wound bacterial clearance time should be measured and recorded for each study group both before and after treatment. If the authors had provided more detailed data on these inflammatory markers, bacterial culture findings, and specific treatment protocols in the article, the overall scientific value of the paper would have been further enhanced.

In conclusion, addressing the aforementioned issues would certainly elevate the overall quality of the article and broaden its appeal to a wider readership.

## Author’s contribution

**Xiuli Li:** Analysis, Writing the manuscript. **Yanwei Sun:** Conception and Design, Writing the manuscript, Final review, Conceptualization.

## Ethical approval

Not required.

## Declaration of competing interest

None declared.
